# Evolution of intersectional perceived discrimination and internalized stigma during COVID-19 lockdown among the general population in Spain

**DOI:** 10.1177/0020764020975802

**Published:** 2020-12-04

**Authors:** Carolina Ugidos, Aída López-Gómez, Miguel Ángel Castellanos, Jesús Saiz, Clara González-Sanguino, Berta Ausín, Manuel Muñoz

**Affiliations:** 1 Chair Against Stigma Grupo 5-Complutense University of Madrid, School of Psychology, Department of Social, Labor and Differential Psychology, Complutense University of Madrid, Madrid, Spain; 2 Chair Against Stigma Grupo 5-Complutense University of Madrid, School of Psychology, Complutense University of Madrid, Madrid, Spain; 3 Chair Against Stigma Grupo 5-Complutense University of Madrid, School of Psychology, Psychobiology and Methodology in Behavioral Sciences Department, Complutense University of Madrid, Madrid, Spain; 4 Chair Against Stigma Grupo 5-Complutense University of Madrid, School of Psychology, Personality, Evaluation and Clinical Psychology Department, Complutense University of Madrid, Madrid, Spain

**Keywords:** Intersectional discrimination, internalized stigma, COVID-19

## Abstract

**Background:**

Stigma and discrimination have been associated with different diseases and pandemics, with negative consequences for the people who suffered them and for their communities. Currently, COVID-19 has become a new source of stigmatization.

**Aims:**

The aim of the present study is to analyze longitudinally the evolution of intersectional perceived discrimination and internalized stigma among the general population of Spain, at three points in time throughout the confinement.

**Method:**

Participants completed an online survey.

**Results:**

Results show an increase in both variables from the first to the second evaluation, and a slight decrease from the second to the third evaluation. Moreover, these changes are explained by depression, anxiety and family support.

**Conclusions:**

These findings indicate the factors that need to be considered to reduce the perception of discrimination and the internalization of stigma, and their detrimental consequences, during an especially stressful event such as the current pandemic outbreak.

## Introduction

The outbreak of COVID-19 has promoted the application of unprecedented measures in many countries. In relation with the evolution of the situation in Spain, the state of emergency was declared on March 14 and drastic isolation measures were applied to all citizens. From March 30 to April 12, all work not considered essential was suspended, aggravating the already existing economic crisis. On May 4, the country began opening up and the lockdown measures were gradually lifted through June 21, when the country began a period called the ‘new normality’. At the beginning of July, more than 250,000 people had been infected in Spain, which was leading Europe in the number of cases, with more than 28,000 deaths (Health Alert and Emergency Coordination Centre, Government of Spain, 2020). The psychological consequences of this situation for the Spanish population include grater psychological distress, PTSD, depressive symptoms, higher levels of stress, anxiety, loneliness, and perceived discrimination (González-Sanguino et al., 2020a, 2020b).

Stigma is a devaluating attribute that has negative connotations for the stigmatized person, producing discredit associated with a disadvantage (Goffman, 1963). Stigmatization often occurs towards certain social minorities, as well as being associated with health problems in diseases that traditionally, mainly due to ignorance, have generated fear and suspicion, such as AIDS or mental health problems (Eaton et al., 2018; Pescosolido, 2013). Stigma can be divided into three components in constant interaction: stereotypes (knowledge structures about people in different groups), prejudice (negative emotions produced when those stereotypes are applied to that group), and discrimination (rejection behaviors directed towards that group) (Ottati et al., 2005). In addition, having multiple identities or social roles can cause intersectional discrimination (Crenshaw, 1989). In other words, the different categories of identity can co-exist and cross over into the same individual, giving rise to an experience (McCall, 2005) with a multiplying effect due to the interaction of the categories. On the other hand, it is also possible to talk about internalized stigma or self-stigma. This concept refers to the stigma that each person feels when internalizing the stereotypes and beliefs about the stigma associated with various conditions (Corrigan & Watson, 2004).

Currently, the recent appearance of the COVID-19 pandemic and the complicated socioeconomic and health situation that it has generated worldwide may be a source of stigmatization, as a certain amount of coronaphobia has already appeared (Asmundson & Taylor, 2020; He et al., 2020). For example, people who have just been diagnosed with COVID-19 may suffer discrimination at the social level and also internalize these beliefs and apply them to themselves (for instance, thinking that the disease is their responsibility or that, because of it, they may be dangerous and rejected). This can generate emotions of self-prejudice, such as feelings of guilt, shame or sadness, which will end up conditioning their behavior.

The effects of discrimination and internalized stigma are numerous, including work stress, mental disorders (Moya & Moya-Garófano, 2020), anxiety, depression, substance abuse (Burgess et al., 2008), and lower self-esteem and wellbeing (Pascoe & Richman, 2009). These consequences may be especially severe in the context of a disease outbreak. As outlined by Brooks et al. (2020) in their review, people who are infected may delay seeking care for fear of being discriminated (Person et al., 2004). Moreover, discriminatory behavior and stigmatization towards health professionals (Desclaux et al., 2017) and minority groups (Pellecchia et al., 2015) was found in previous epidemics. In the COVID-19 context, Singh and Subedi (2020) note that not only those patients that currently have COVID-19 and healthcare providers, but also those who have recovered from the disease are facing discrimination. In some cases, they have been denied entrance to communities for fear of transmitting the virus to others. In addition, it should be noted that political leaders have misappropriated the COVID-19 crisis to reinforce racial discrimination (Devakumar et al., 2020).

Considering the consequences, it is important to know which factors influence these variables. Among the psychosocial variables found to be related to stigma and discrimination, social support appears to be particularly relevant, especially due to the isolation caused by the lockdown measures adopted to prevent the spread of COVID-19. Social support has shown to be a protective variable against the effects of discrimination for different groups (Cristini et al., 2011; Seawell et al., 2014), and even in the context of this pandemic it has demonstrated that it reduces the psychological impact of this stressful situation (Lei et al., 2020). Similarly, in Spain, previous research has shown that relationship between perceived discrimination and social support in a sample of family caregivers of children with intellectual disabilities (Recio et al., 2020), and between internalized stigma and support from friends, coworkers, and health care providers in people living with HIV (Garrido-Hernansaiz & Alonso-Tapia, 2017). Furthermore, several studies point to the effect of discrimination and stigma on depression and anxiety (Burgess et al., 2008; Moya & Moya-Garófano, 2020). For instance, in Spain, this association has been found in people with obesity (Magallares et al., 2017), in people with dwarfism (Fernández et al., 2012), and in people with schizophrenia (González et al., 2018). Research, however, does not usually focus on the effect that cognitive biases produced by depression and anxiety (Beck, 2008) could have on the perception of discrimination and the internalization of stigma.

Although several scientific articles have drawn attention to the possible increase of stigma and discrimination due to COVID-19 (Asmundson & Taylor, 2020; Devakumar et al., 2020; Logie & Turan, 2020; Singh & Subedi, 2020; Teixeira da Silva, 2020; Zhai & Du, 2020), only one study has been published that assesses the impact of the pandemic and the resulting crisis situation on discrimination and stigmatization of persons of Chinese nationality across 70 countries (He et al., 2020). The findings show that 25.11% of participants reported to have experienced different forms of discrimination. Women, young people and those who are less educated are more likely to experience discrimination and even violent overreactions, while people with permanent resident status are less likely to report such experiences. Interestingly, respondents living in countries with a high number of confirmed cases of COVID-19 are less likely to report cases of discrimination and overreaction. Social stigma reduces the likelihood that infected people will come forward for help, preventing medical practitioners from effectively containing and treating the disease in the early stages.

To our knowledge, no longitudinal studies have been published assessing discrimination and internalized stigma during the state of alarm declared to contain COVID-19. The present study aims to conduct a longitudinal analysis of the evolution of intersectional perceived discrimination and internalized stigma among the general population of Spain at three points in time: 2 weeks after the beginning of the confinement, 1 month after the beginning, and 2 months after, when the country began lifting restrictions and returning to the ‘new normality’.

## Method

### Procedure

The longitudinal study took place between March 21 and 29 (first evaluation), between April 13 and 27 (second evaluation) and between May 21 and June 4 (third evaluation). Data was collected online through Google Forms in an attempt to reach the maximum population possible. The first survey consisted of 80 items (15 minutes long). At the end of the questionnaire, a section was included describing the research, as well as the consent form to participate in the study and acceptance of the data protection laws regarding the regulation (EU) 2016/679 of the European Parliament and of the Council, of 27 April 2016, on the protection of personal data. Participants were given the possibility of completing the second and third evaluation. Those who agreed received the survey via email during the second and third data collection periods.

### Participants

The sample was recruited by sending requests for participation to people belonging to databases of different institutions: students and workers in public organizations, such as Complutense University of Madrid and the Chair for Stigma, and private organizations, such as the company Group 5. These databases are complete enough to make a reasonable sampling of the Spanish population. To increase the sample size as much as possible participants were asked to help with its dissemination. The percentage of people recruited in this way was small, estimated at less than 5%. The sample of the first evaluation had 3,480 participants, made up of the general population. Participants were given the opportunity to take part in subsequent surveys by providing their email on the first questionnaire. After contacting all the participants who agreed to be part of the second evaluation, 1,041 people answered the second questionnaire. Similarly, 568 people participated in the third and last survey. The inclusion criteria for the three rounds were: to be over 18 years of age, and to be living in Spain during the COVID-19 state of emergency. In the resulting sample, a majority of women (81%) was obtained as opposed to 51% of the general population. With respect to age, a greater equivalence was obtained, although with a higher percentage of people under 60 years than in the general population: 29% (18–30), 64% (31–59), and 7% (60–80) for the three respective groups, compared to 10%, 44%, and 19% for the general population (the remaining 5% do not meet the criteria for inclusion/exclusion). The influence of these differences is discussed in the discussion section.

### Variables and instruments

The following variables and instruments were included in the assessment:

#### Sociodemographic variables

Using ad hoc questions, data was collected on age (subsequently grouped into clusters: 18–30, 31–59, 60–80); gender identity; marital status (single, married, divorced, separated, widower); educational level (elementary studies, high school, vocational training, university, postgraduate); economic situation (subjective perception from very bad to very good).

#### COVID-19 related variables

Suffering from symptoms (yes, no); existence of a family members or close relatives who are infected (yes, no); perception of the information received on the alarm situation (considering that they have sufficient information, or that they are over-informed).

#### Intersectional discrimination

Intersectional discrimination was evaluated by means of the Intersectional Day-to-Day Discrimination Index (InDI-D) (Scheim & Bauer, 2019), in its Spanish version, which was translated by the authors of this study. This scale provides a measure of the intersectional discrimination that can be produced by different conditions: gender, ethnicity, mental health diagnosis, and in this case, the presence of COVID-19 was also included. We used the main scale formed by 9 Likert-type items (e.g. ‘Since the sanitary emergency caused by COVID-19 in Spain, have you been treated as if you were someone hostile, unhelpful or rude?’) with four response options (1 ‘never’ – 4 ‘many times’). The different questions evaluated the presence of intersectional discrimination from the beginning of the alarm situation generated by the coronavirus. The higher the score the more discrimination suffered. The adjusted ICC for test-retest reliability of the original version was 0.70 (95% CI: 0.62, 0.78). For the Spanish version, the scale’s consistency was adequate (α = 0.76).

#### Internalized stigma

Internalized stigma was evaluated with two items adapted from the Internalized Stigma of Mental Illness (ISMI) scale (Boyd Ritsher et al., 2003). The items (‘Since the emergency situation generated by the coronavirus, have you avoided contacting people – in those cases permitted during lockdown – to avoid rejection?’; ‘Since the emergency situation generated by the coronavirus, have you felt that the people who are not in your situation are unable to understand you?’) were modified to evaluate intersectional internalized stigma, the self-stigma that can be generated by diverse conditions. These items refer to the alienation and social withdrawal dimensions taken from the original scale. It was evaluated with the same Likert-type scale as the one used to measure the intersectional perceived discrimination.

#### Social support

Social support was evaluated by means of the Multidimensional Scale of Perceived Social Support (EMAS) (Zimet et al., 1988), adapted to a Spanish version (Landeta & Calvete, 2002). The scale, made up of 12 Likert-type items with 7 possible responses (1 ‘totally disagree’–7 ‘totally agree’), evaluates the levels of perceived social support, identifying where the support comes from and how it is perceived. The EMAS explores three possible sources of perceived social support: family (4 items), friends (4 items), and relevant people (4 items), and offers a full measure of social support. Cronbach’s α is 0.89 for the Spanish version.

#### Mental health

Mental health was assessed with the PHQ-4 composed by the Patient Health Questionnaire 2 (PHQ-2) (Kroenke et al., 2009) and the Generalized Anxiety Disorder Scale (GAD-2) (Spitzer et al., 2006). The PHQ-2 was used in its Spanish version (Diez-Quevedo et al., 2001) and is a brief self-report questionnaire that addresses the frequency of depressive symptoms. It consists of 2 Likert-type questions ranging from 0 ‘never’ to 3 ‘every day’. Higher scores indicate greater symptomatology, providing a severity score that ranges from 0 to 6. A score of >3 points was established as the cut-off point indicating a possible case of depression (Muñoz-Navarro et al., 2017). The original scale presented a sensitivity of 0.9 and a specificity of 0.61 (Kroenke et al., 2009). GAD-2 was also used in its Spanish version (Garcia-Campayo et al., 2014). The GAD-2 Questionnaire includes the first two items of the GAD-7 Likert format, with a maximum score of 6 points. The cut-off point in this case is 3, above which possible anxiety is indicated (Muñoz-Navarro et al., 2017). The sensitivity of the original test was 0.88, with a specificity of 0.61.

### Analysis

To analyze the effect of longitudinal measures, linear mixed models were calculated for perceived discrimination and internalized stigma. As data contain missing values (participants who did not respond to successive surveys), the random effects were calculated as random slopes (without random intercepts) so that the models could be estimated. The predictor variables that varied across time were considered as non-correlated. The results include the value of Nakagawa’s Psuedo-R2 (both marginal and conditional). The marginal R2 considers exclusively the variances of the fixed component while the conditional R2 considers both the fixed and random effects. Moreover, post hoc comparisons were calculated using the estimated marginal means with the Tukey adjustment. The analyses have been performed using R (v3.5.6) with the lme4 and emmeans packages.

The study was approved by the Deontological Commis-sion of the Faculty of Psychology of the Complutense University of Madrid with reference ‘pr_2019_20_029’.

## Results

### Sociodemographic and COVID-19 data

The sample is mostly formed by women (80%), people aged between 31 and 59 years (64%), those who are single (52%), have a university degree (38%) and a good or very good perceived economic situation (60%). Regarding COVID-19 variables, the majority of the participants did not have symptoms of COVID-19 (80%), nor a diagnosed relative (70%), and most of them considered they have received enough information about this disease (58%). The percentage of these variables remains fairly stable across the three evaluations. This information can be found in Table 1.

**Table 1.** Sociodemographic and COVID-19 data.

	T0	T1	T2
	*N* (%)	*N* (%)	*N* (%)
Gender			
Female	2,584 (75%)	841 (81%)	453 (81%)
Male	860 (25%)	202 (19%)	104 (19%)
Age			
18 to 30	1,216 (35%)	306 (29%)	148 (27%)
31 to 59	2,035 (59%)	670 (64%)	364 (65%)
60 to 80	200 (6%)	69 (7%)	46 (8%)
Marital status			
Single	1,900 (55%)	542 (52%)	268 (48%)
Married	1,231 (36%)	386 (37%)	227 (41%)
Divorced	214 (6%)	82 (8%)	42 (8%)
Separated	67 (2%)	28 (3%)	17 (3%)
Widower	39 (1%)	7 (1%)	4 (1%)
Education			
Elementary	98 (3%)	15 (1%)	6 (1%)
High school	599 (17%)	149 (14%)	69 (12%)
Vocational training	439 (13%)	125 (12%)	68 (12%)
University	1,294 (37%)	401 (38%)	216 (39%)
Postgraduate	1,021 (30%)	355 (34%)	199 (36%)
Perceived economic situation			
Bad-very bad	348 (10%)	111 (11%)	58 (10%)
Good-very good	1,975 (59%)	621 (60%)	359 (65%)
Neither good nor bad	1,042 (31%)	304 (29%)	137 (25%)
COVID-19 symptoms			
No	2,974 (86%)	836 (80%)	445 (80%)
Yes	477 (14%)	209 (20%)	113 (20%)
COVID-19 diagnosis for a relative			
No	2,474 (72%)	638 (61%)	380 (68%)
Yes	977 (28%)	407 (39%)	178 (32%)
Information received about COVID-19			
Insufficient	614 (18%)	184 (18%)	96 (17%)
Good	1,983 (57%)	594 (57%)	326 (58%)
Over-informed	854 (25%)	267 (26%)	136 (24%)

### Longitudinal changes on intersectional discrimination and internalized stigma

**Figure 1.**
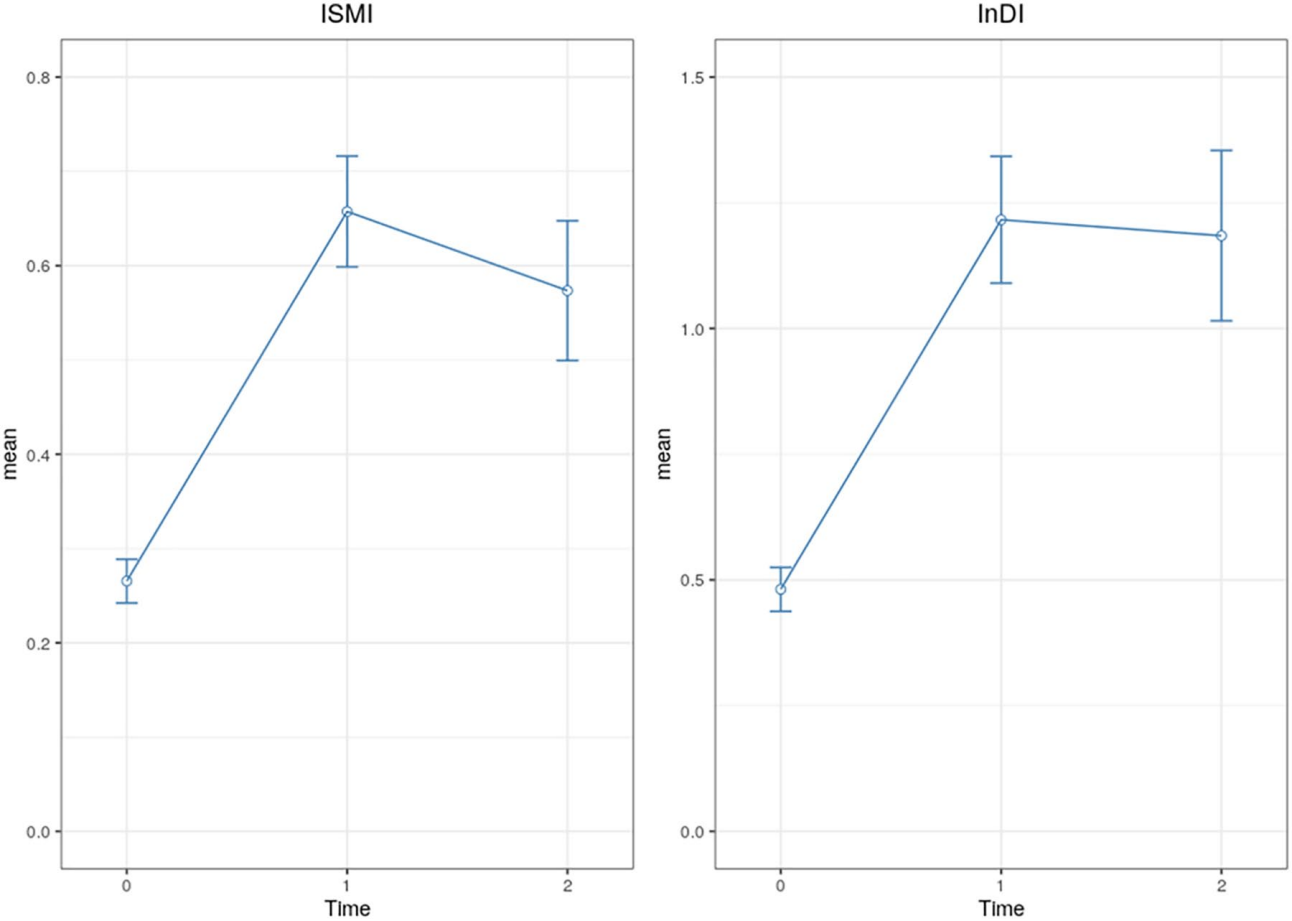
Longitudinal changes on intersectional perceived discrimination and internalized stigma.

As shown in Figure 1, from the first to the second evaluation, results show a significant increment in intersectional discrimination (Z(T0-T1) = 15.02, *p* < .001) and internalized stigma (Z(T0-T1) = 16.27, *p* < .001). However, there is a small decrease in internalized stigma (Z(T1-T2) = 2.36, *p* = .047) between the second and third evaluation, while the difference in intersectional discrimination is not significant (Z(T1-T2) = 0.34, *p* = .936).

### Linear mixed models

The model for intersectional discrimination explains 10% of the variance of the fixed effects, with depressive and anxious symptomatology and less family support as the main predictors. These results can be observed in Table 2. On the other hand, the model for internalized stigma, as shown in Table 3, explains 14% of the variance of the fixed effects, also with depressive and anxious symptomatology and less family support as the main predictors.

**Table 2.** Linear mixed model for intersectional perceived discrimination.

Fixed effects: mean sq		*df*1	*df*2	*F*	*p*
Time	189.52	1	1,027.9	107.57	<.001***
PHQ4	353.35	1	4,642.3	200.57	<.001***
SS-family	194.61	1	4,707.1	110.46	<.001***
Random effects			Pseudo-*R*^2^		
Time|id	0.131		Conditional	0.341	
Residual	0.458		Marginal	0.108	

* *p* < 0.05, **p < 0.01, ***p < 0.001.

**Table 3.** Linear mixed model for internalized stigma.

Fixed effects: mean sq		*df*1	*df*2	*F*	*p*
Time	58.15	1	1,048.7	126.73	<.001***
PHQ4	174.73	1	4,816.5	380.82	<.001***
SS-family	45.04	1	4,870.0	98.15	<.001***
Random effects			Pseudo-*R*^2^		
Time|id	0.131		Conditional	0.280	
Residual	0.458		Marginal	0.144	

* *p* < 0.05, z ** *p* < 0.01, ***p < 0.001

## Discussion

This is the first longitudinal study that analyzes the evolution of intersectional perceived discrimination and internalized stigma among the general population of Spain. The results show their evolution during the confinement period, and the variables that influence them. Specifically, the findings obtained indicate the effect of mental health and family support on the development of both dependent variables.

From the first to the second evaluation, results show a significant increase in intersectional discrimination and internalized stigma. However, there is a small decrease in internalized stigma between the second and third evaluation, while the difference in intersectional discrimination is not significant. These results can be explained by the fact that the first data collection took place when the increase of COVID-19 infections among the Spanish population started. During the first month of confinement the number of COVID-19 infections increased exponentially, which may have caused more people to experience discrimination for being infected or for other reasons. These could include loss of employment, the need for many people to stay at home and give up a job to be able to reconcile caring for children and other family members, living in places with a high percentage of infected people (such as Madrid or Catalonia), being a worker at high risk of infection such as healthcare professionals or supermarket cashiers, among others. It should also be noted that although there are no pre-pandemic measures on stigma, the trends found show that discrimination and internalized stigma increase with the evolution of the crisis, decrease with the beginning of recovery and return to normal, although without returning to previous levels.

The variables that best predict perceived intersectional discrimination and internalized stigma are depression and anxiety, and less family support. These results could be explained by the fact that family support is a protective variable, allowing people to feel included in a family nucleus. Social support can buffer the harmful effects of stressful events by providing a sense of acceptance and self-worth (House, 1981), and thus reducing internalized stigma. Similarly, family support could influence the appraisal of stigmatizing events (Aspinwall & Taylor, 1997), decreasing the perception of discrimination. Only family support, not support from other sources, has an impact on these variables. This might occur due to the confinement. During this period people could only interact in person with the people living with them, who, in most cases, are their relatives. Several studies have shown the protective effect of social support for different groups of people, such as immigrant adolescents or African-American women (Cristini et al., 2011; Seawell et al., 2014). Likewise, Ahuja et al. (2020) found collectivistic tendencies (feeling of belongingness, greater strength of social connections and importance given to needs of one’s family) buffer the levels of uncertainty and stress caused by this infectious disease. Other studies also point to the reduced impact of psychosocial stressors on individuals with better social support from their family and social networks (Lei et al., 2020). In order to mitigate the effects of social isolation, in Spain, mutual support networks have been activated in various neighborhoods across the country. This is not new in the Spanish background, in which the neighborhood and its associative fabric became an agent of resistance against the vulnerabilities produced by the 2008 crisis (Cano-Hila & Argemí-Baldich, 2020). In this context, the neighborhood is understood as a space for strengthening social capital, solidarity, community building, and social cohesion (Blokland, 2017; Kennett & Forrest, 2006).

As for the variables of depression and anxiety, previous research conducted in Spain has found how discrimination was related to a greater psychological impact (González-Sanguino et al., 2020a, 2020b). In this regard, some authors such as Beck (2008) explain that depressed patients show a tendency to develop highly dysfunctional attitudes that can ‘misappropriate’ information processing by producing cognitive biases. Similarly, in the research carried out by Caouette and Guyer (2016), the relationship between depression and emotional responses of social acceptance and rejection was studied. The results showed that depression interfered through attenuated cognitive response to social acceptance and rejection. In other words, cognitive biases seemed to contribute to this emotional insensitivity context. Thus, ‘the individual affectively “disengages” from valenced social feedback in anticipation of harmful outcomes’. These biases could explain the greater perception of discrimination, and consequently, the internalization of the stigma.

Various agencies and scientific publications have made recommendations and launched campaigns to combat the stigma associated with the pandemic (IFRC, UNICEF, & WHO, 2020; Singh & Subedi, 2020) In general, recommendations and actions taken often stress the importance of being careful of the language used when talking about the disease, avoiding the spread of false news and being careful with communication, disseminating precise information related to COVID-19 to the public, facilitating the request for help and, in general, providing comprehensive support to frontline healthcare providers both from administrators and society. This is in line with the Health Stigma and Discrimination Framework, which posits once stigma is applied to people with a specific disease, such as COVID-19, interventions have to shift harmful attitudes and behaviors that compromise the health and wellbeing of affected communities (Stangl et al., 2019). Furthermore, based on the findings attained in this study, it would be recommendable to enable the creation of support networks (through online means if there are mobility restrictions), especially for people who are not living with their families during the confinement, and facilitate the access to psychological treatment for depression and anxiety.

As limitations of this study, we include the loss of participants throughout the assessments, especially in the third evaluation, which may be a sign of a return to normality and loss of interest in the phenomenon. Moreover, as indicated in the participants section, despite the effort in recruitment, the resulting sample is not exactly equivalent to the Spanish population. This fact does not distort the results found, since the objective is not to provide epidemiological information or prevalence data but to compare the averages obtained by various social groups in the variables of interest and to analyze the differential change between temporal measures. In this sense, as long as the sample meets the requirements of the statistical tests used, we believe it is valid for the study. However, it is necessary to be careful in the interpretation of the results and understand that they are limited by the characteristics of the sample obtained.

Despite these limitations, this is the first longitudinal study analyzing the evolution of intersectional perceived discrimination and internalized stigma during a pandemic outbreak. The results presented show new consequences derived from the pandemic related to the phenomenon of stigmatization, and remind us of the need to address this phenomenon by understanding its key variables.

In conclusion, the findings obtained in this study have important implications in the developing of effective strategies to tackle the study variables. More specifically, it is necessary to reduce depression and anxiety, and boost family support in order to buffer the perception of discrimination and internalization of stigma, and thus their detrimental consequences.
